# Public Awareness and Practices towards Self-Medication with Antibiotics among the Malaysian Population. A Development of Questionnaire and Pilot-Testing

**DOI:** 10.3390/antibiotics9020097

**Published:** 2020-02-24

**Authors:** Adeel Aslam, Márió Gajdács, Che Suraya Zin, Norny Syafinaz Binti Abd Rahman, Syed Imran Ahmed, Shazia Qasim Jamshed

**Affiliations:** 1 Department of Pharmacy Practice, Kulliyyah of Pharmacy, International Islamic University Malaysia, Kuantan, Pahang 25200, Malaysia; adeelaslam224@gmail.com (A.A.); chesuraya@iium.edu.my (C.S.Z.); norny@iium.edu.my (N.S.B.A.R.); 2 Department of Pharmacodynamics and Biopharmacy, Faculty of Pharmacy, University of Szeged, 6720 Szeged, Hungary; gajdacs.mario@pharm.u-szeged.hu; 3 Institute of Medical Microbiology, Faculty of Medicine, Semmelweis University, 1089 Budapest, Hungary; 4 Department of Pharmacy Practice, School of Pharmacy, International Medical University, Kuala Lumpur 57000, Malaysia; imran_ahmed@imu.edu.my; 5 Qualitative Research-Methodological Application in Health Sciences Research Group, Kulliyyah of Pharmacy, International Islamic University Malaysia, Kauantan, Pahang 25200, Malaysia

**Keywords:** self-medication, antibiotics, reliability, validity

## Abstract

It is well documented that injudicious antibiotic use and practicing self-medication with antibiotics (SMA) can lead to antibiotic resistance. The objective was to validate and develop an instrument in Bahasa Melayu to assess the awareness and practices towards SMA in the Malaysian population. A pilot study was conducted among 100 Malaysians participants. Reliability testing in terms of test-retest, internal consistency, and content validity was performed. One-way ANOVA and t-test were applied to determine significant differences between groups. A panel of nine experts evaluated the research instrument for content validity and it was found to have strong content item validity (Indices = 1). Each domain (level of knowledge and understanding about antibiotic use and antibiotic resistance: Practice towards self-medication) showed good internal consistency of Cronbach’s alpha 0.658 and 0.90. While test-retest reliability value for each domain was 0.773 (*p* = 0.009), and 0.891 (*p* = 0.001. The mean ± standard deviation (SD) for level of knowledge about antibiotic use and antibiotic resistance was 21.8 ± 7.02 and for practice scores (SMA) 6.03 ± 2.30. The instrument established sound reliability and validity and, therefore, can be an effective tool for assessing public awareness, and practices toward self-medication with antibiotics in the Malaysian population.

## 1. Background

Around 80% of antibiotics are utilized among the community, the remaining are utilized inside hospitals [1,2], and 20–50% of all antibiotics are utilized inappropriately [2]. Self-medication with antibiotics (SMA) is defined as the utilization of medications to take care of a self-diagnosed disease, or signs or symptoms, or the sporadic or extended usage of an approved medicine for long-term use, or for persistent disease or symptoms [3]. SMA makes up a significant form of aberrant usage of medication and could propose significant undesirable effects, for example, drug toxicity, resistant microorganisms, continued hospitalization intervals, treatment failures, increase in treatment price, and an upsurge in the morbidity rate in the community [4,5,6]. Furthermore, antibiotics is one of the most commonly prescribed medicines worldwide, and because of this antibiotic’s resistance, it is without a doubt a first-rate public health issue.

According to a report developed by WHO, the SMA is generally widespread in the developing countries [6], and therefore, it is necessary to analyze general public knowledge and understating about antibiotic utilization patterns, which can help concerned authorities develop ideal interventions programs against SMA. The recurrent utilization of SMA inside developing countries is related to several elements, particularly insufficient access to healthcare, accessibility to antibiotics sold as per over-the-counter drugs, deprived regulatory strategies, and higher frequency of contagious diseases as compared to developed countries [7,8,9]. Laws pertaining to drug regulations that influence the availability of medication are executed differently within the countries, and play a crucial role in misconceptions regarding the use of antibiotics [10]. Furthermore, regulation enforcement regarding antibiotics dispensing varies among countries. For example, common practice towards SMA in Spain may result in consequences that involve little enforcement, and control over the laws and regulations affecting prescription, which have a knock-on effect upon community pharmacy services [11]. 

In Southeast Asia, the upsurge in economic growth led to expanded utilization of antimicrobials, and Malaysia is not an exception in this regard. In Malaysian Statistics on Medicine 2009–2010, the Ministry of Health reported a 16% increased use of both prescribed and purchased over-the-counter antimicrobials annually, and this can contribute to antimicrobial resistance. Self-medication practice is a worldwide predicament, and those who practice self-medication are ignorant of the adverse consequences, such as antimicrobial resistance, and even symptom aggravation. The global evidences speak volumes of the involvement of the lay public, predominantly in this practice, and therefore the current research in the Malaysian lay public can be instrumental in highlighting their awareness of and practices toward antibiotic use and antimicrobial resistance. Furthermore, one in five patients received antimicrobial prescription from a primary healthcare center. Moreover, the prevalence of self-medication with antibiotics is increasing gradually; therefore, the development of a valid measure encompassing the awareness and knowledge of the population regarding antibiotic use, as well as self-medication practices with antibiotics, is undoubtedly a valuable effort for its use in research and clinical practices. Furthermore, keeping this in context, the need for reliable and valid instruments in the Malay language is impactful, because this allows for comparison with international literature. In a nutshell, the current research is of high relevance for both the advancement of clinical practices and better patient outcomes.

## 2. Aims and Objectives

The aims and objectives of the current study was to develop a questionnaire to measure general public awareness and practices towards SMA among the Malaysian population, and for this translation and development of a questionnaire/instrument from English to Malay is necessary. Reliability, as well as validity of the translated questionnaire/instrument, was established and variations in practices towards SMA among socio-demographic characteristics of the Malaysian population also assessed. The final version of the questionnaire/instrument will also help identify the most common determents among the Malaysian population regarding SMA. 

## 3. Methods

### 3.1. Questionnaire Development

The questionnaire is used for data collection related to healthcare research [12,13,14,15]. The initial questionnaire originated in the English language, and in order to maintain uniformity among the questions, adapted with and without changes.

### 3.2. Process of Adaptation and Translation of Questionnaire

For the purpose of adaptation and translation of the questionnaire, a set criterion was followed, developed by Beaton and Guillemin [16,17]. In the initial step, two distinct interpreters/translators, whom equally speak Bahasa Melayu as well as English (but their native or inborn language was Bahasa Melayu), translated the questionnaire from English into Bahasa Melayu. Furthermore, to improve the quality of translated instruments, among the two translators, one was aware with the aims and objectives of the questionnaire, whereas the other had not been aware of the aims and objectives. When the first edition of the Malay questionnaire was ready, one Malaysian-Malay analyzed or compared the Malay version with the original version.

In the next step, reverse translation of the questionnaire/instrument was done from the Bahasa Melayu version back into the English version. The reverse or back translation was completed by two additional interpreters/translators who were equally fluent in both Bahasa Melayu and English, but both translators were not aware of the aims and objectives of the questionnaire. After the completion of the reverse translation of the Malay version questionnaire back into the English version, was compared to the original English questionnaire, and the subsequent report was prepared. In order to ensure accuracy, the questionnaire’s repetitive discussions section was carried out among the researchers and translators.

### 3.3. Pilot Test of the Questionnaire

In the last stage of questionnaire development, a pilot testing was performed. It was also very essential to ensure that appropriate data collected for pilot testing during the development of a final questionnaire. The purpose of pilot testing is to identify such items that lack clarity among respondents. The final version of the questionnaire’s (Bahasa Melayu) edition on awareness and SMA was carried out and prepared for the reliability and validity analysis. 

### 3.4. Data Collection

The final data collection form, consisted of five different parts, and the initial part contained the individual’s socio-demographic information. The next component consisted of 12 statements; this part was designed to collect data about personal information on each participant’s health and medicine use. Respondents were further asked about reasons, why they didn’t visit a doctor the last time when needed, and common sources from where they buy antibiotics. While the third part of the questionnaire comprises of 31 statements to assess the level of knowledge and understanding about antibiotic use and antibiotic resistance. The fourth part of the questionnaire consists of three questions (10 statements) on practices towards SMA, and in this domain, respondents were asked to answer questions about common elements related to SMA, and most common sources, and frequently used antibiotics at the time of the cold, flu, sore throat, and diarrhea. Participants, who were aged between 21 and 64, and who did not have any medical background, were included in this study. The study site was Kuala Lumpur, the biggest global city of Malaysia, and covers an estimated area of 243 km^2^ with a population of 1.73 million, according to a census carried out in 2016 [18]. The data had been gathered from the general public across the city of Kuala Lumpur. For the pilot testing of the questionnaire, the recommended sample size was less than 100 subjects [19]. Before the commencement of the study, the ethical approval was taken from the Research Ethics Committee (IREC) of the International Islamic University Malaysia. An information sheet describing aims and objectives was attached to every questionnaire, and an informed consent form was also obtained from each participant, after the objective and procedure of the study was explained. 

### 3.5. Demonstrating Reliability 

Reliability identifies the internal consistency as well as repeatability among questions [20]. So, it is crucial that the reliability of the questionnaire must be established. Among the most common methods that demonstrate reliability is the use of Cronbach’s α statistic. Cronbach’s α statistic is used to find out inter-item correlations, to determine if the fundamental questionnaire is calculating the same domain or not [20,21,22]. To determine the reliability among the questions, internal consistency reliability tests, as well as test-retest reliability, were executed. For the test, re-test data was collected from a subgroup of 10 individuals through the final version of the questionnaire, and after a period of two-weeks; again, data was collected from the same individuals. To construct validity of the questionnaire, and to assess the level of knowledge, and understand practices towards SMA through known-groups, validity method approach was used in this study.

### 3.6. Demonstrating Validity

A panel of nine experts, who are specialists in pharmacy practice fields, discussed and appraised both content and face validity for the final version of the questionnaire. The content validity index (CVI) for questions had been calculated and modified as required.

### 3.7. Statistical Analysis

The data was collected and analyzed by utilizing Statistical Package for Social Sciences (SPSS) version 22 for Windows. The frequency as well as percentage of every demographic were evaluated. Furthermore, mean and median values were calculated for total level of knowledge scores and practice (with SMA) scores. Scoring of the questions was determined by giving one point (1) for each correct answer and zero (0) for incorrect answers or for no response. The level of significance was set at *p* < 0.05 throughout data analyses, and normality of continuous data was determined through the Skewness and Kurtosis test. Reliability for the questionnaire was tested for both internal consistency as well as corrected item-total correlations through Cronbach’s alpha coefficient. Pearson correlation coefficient was used to obtain test-retest reliability values [19]. While, known group validity was evaluated through the association of demographic parameters, with total knowledge score and total practice scores related to SMA through one-way ANOVA and an independent *t*-test.

### 3.8. Ethical Approval 

The study was approved by the Research and Ethics Committee of the International Islamic University Malaysia. (Reference number, IIUM/504/14/11/ IREC 2019-004).

## 4. Results

### 4.1. Demographic Characteristics

Among the 100 participants, there were adults in the age range between 21 and 60 years (mean = 32.89, SD = 8.61) with 48% being male and 52% female. The majority of participants were Malay (40%), and most of the participants had a secondary school degree (39%) (Table 1, Table 2, Table 3 and Table 4).

### 4.2. Reliability

#### 4.2.1. Internal Consistency

The results of internal consistency analysis for each dimension are presented in Table 5, Table 6 and Table 7. Internal consistency was determined for knowledge as well as for understanding about antibiotic use and antibiotic resistance and has Cronbach’s alpha value 0.658. While practice towards self-medication and patient-reported outcomes Cronbach’s alpha value 0.90 and 0.670 respectively.

#### 4.2.2. Test-Retest Reliability

Pearson’s correlation coefficient was applied to investigate test-retest reliability, and results indicate satisfactory reliability and stability. For domain knowledge and understanding, practice towards SM, and patient-reported outcomes were 0.773, 0.891, and 0.787 respectively (*p* < 0.05).

### 4.3. Association of Demographic Characteristics of Participants with Total Level of Knowledge and Understanding Scores about Antibiotic Use and Antibiotic Resistance

ANOVA and an independent t-test revealed differences among demographic characteristics. Results showed that participants’ total knowledge and total understanding scores differ significantly with respect to gender (*p* = 0.001): males have low total knowledge and total understanding scores about antibiotic use and antibiotic resistance as compared to females. A significant difference was also found in total knowledge and understanding scores between participants’ education (*p* = 0.001), participant duration of stay in Malaysia (0.003), and in ethnic groups (*p* = 0.004). While the age group, marital status, place of birth, and income showed no significant difference (Table 8).

### 4.4. Association of Demographic Characteristics with Total Practice Scores for SMA

Results demonstrate that total practice scores for SMA differ significantly with respect to gender (*p* = 0.004), where females are more prone to do SMA as compared to males. A significant difference was also found in total practice scores for SMA among household income (*p* = 0.005) and education (*p* = 0.002). While the age group, marital status, place of birth and occupation showed no significant difference. Participants with higher education (post-graduation) and with lower income doing more antibiotics, as compared to others (Table 9).

## 5. Discussion

The current global scenario of antimicrobial resistance paves the way for addressing the understanding and knowledge of antibiotic use, and self-medication practices towards antibiotics, especially among the lay public, who are reported to be rampant consumers of injudicious antibiotics. Previously, few scales have been developed in different regions, but they are either not fully validated or developed only for healthcare professionals and future healthcare practitioners.

This current study was aimed to develop a valid questionnaire to effectively measure general public awareness and practices towards SMA among the Malaysian general public. The original questionnaire was systematically developed for the precise context in the English language and then translated in measurable terms to the local Malaysian language by following the guidelines of Beaton (2000) and Guillemin (1993). Content validity ensures the quality of the questionnaire [23] and in this study face, validity and content validity was assessed by a panel of nine experts with the background of pharmacy practice, and item validation results demonstrate that the value of CVI is 1. The finalized questionnaire showed good internal consistency reliability, Cronbach alpha, as well as test re-test value for knowledge and understanding about antibiotic use, practice towards SMA, and patient report outcomes. The final research questionnaire was like previous studies performed in different countries, in terms of measuring level of knowledge, understanding and common practices SMA [24,25,26,27]. Hence, results obtained from this study showed that developed questionnaire effectively measure the general public level of knowledge and understanding about antibiotic use, antibiotic resistance, as well as practice towards SMA.

Known group validity showed that females had better knowledge and understanding scores about antibiotic use and antibiotic resistance as compared to males. There was a significant difference noted among participants having higher education and among ethnic groups. Malay ethnicities showed good knowledge and understanding scores when compared to other ethnic groups, and results were similar with another study performed in the state of Penang [26]. This difference might be due to the reason that most of the Malay participants have better education backgrounds as compared to others. Household monthly income also has a positive correlation (*p* = 0.005) with total practice scores for SMA, but no significant difference (*p* = 0.285) was noted with total knowledge and understanding scores about antibiotic use and antibiotic resistance. Gender has significant impact on practice towards SMA, and results were in concordance with other studies performed in Sudan [28]. Others factors, identified from results related to SMA, were low income and education levels (participants with higher education are more prone towards SMA) and results are similar to other studies performed in Jordan and the United Arab Emirates [29,30]. However, the result showed no significant differences in other demographic parameters, such as age, place of birth, marital status, race, duration, of stay occupation.

This questionnaire development study was conducted in Kuala Lumpur and has several limitations. Data were collected during the day in different public places, including malls, supermarkets, and parks, where young male and female abundance is mostly higher at this time. Thus, a higher number of young participants were included in this study. This small number of participants cannot be representative of the whole population. Moreover, urban population was only addressed, and therefore evaluation among the rural lay public is recommended. Furthermore, the questionnaire was not validated against any clinical measure of antibiotic use. Likewise, this sample size is not credible enough to proceed with construct validity; thus, confirmatory factor analysis is another recommended future strategy.

## 6. Conclusions

The developed questionnaire for this pilot study was a valid and reliable instrument for assessment of public awareness and practices towards self-medication with antibiotics. The current questionnaire can serve as a useful tool in research to measure knowledge and practices of the general population towards SMA, and can help government agencies and healthcare professionals toward the development of an effective educational intervention to improve general public health and wellbeing. The comprehensive nature of the questionnaire highlights that it can be safely used in rural populations. To conclude, the study shows encouraging findings, and generates testimony of robust assemblage of conceptually standardized elements.

## Figures and Tables

**Table 1:** Socio-demographic characteristics of study participants.

Variables	Frequency	Percentage
Gender		
Male	48	48%
Female	52	52%
Age (Years)		
21–25	24	24%
26–30	24	24%
30–40	26	26%
Above 40	26	26%
Race		
Malay	40	40%
Chinese	20	20%
Indian	20	20%
Others	20	20%
Place of Birth		
Born in Malaysia	43	43%
Not Born in Malaysia	57	57%
How long you lived in Malaysia		
Local resident	44	44%
0–1 years	3	3%
2–5 years	17	17%
6–10 years	20	20%
Above 10 years	16	16%
Education		
Primary school	6	6%
Secondary school	39	39%
Certificate/Diploma	17	17%
Undergraduate degree/Bachelor degree	33	33%
Postgraduate education	5	5%
Marital status		
Single	38	38%
Married	54	54%
Divorced or Separated	2	2%
Widow	4	4%
Live in relationship	2	2%
Children		
No	51	51%
Yes	49	49%

**Table 2:** Socio-demographic characteristics of study participants.

Variables	Frequency	Percentage
Household monthly income		
Under 3000 Rm	39	39%
3000–5000 Rm	30	30%
Over 5000 Rm	31	31%
Occupation		
Professional	11	11%
Skilled labor	13	13%
Manual labor	15	15%
Administrative	12	12%
Self-employed	24	24%
Home duties	5	5%
Unemployed	17	17%
Pensioner	1	1%
Others	2	2%
Language		
Bhasa Melayu	39	39%
English	10	10%
Mandarin	20	20%
Cantonese	Nil	Nil%
Hokki	1	1%
Tamil	20	20%
Others	20	20%
How good you speak English		
Very good	14	14%
Good	34	34%
Average	39	39%
Poor	8	8%
Very poor	5	5%
Part 2. Personal information on health and medicine use		
Smoke		
Every day	20	20%
3–5 times per week	7	7%
1–2 times per week	4	4%
Not at all	69	69%
Drink alcoholic beverage		
Every day	1	1%
3–5 times per week	3	3%
1–2 times per week	8	8%
Not at all	88	88%

**Table 3:** Socio-demographic characteristics of study participants.

Variables	Frequency	Percentage
Exercise		
Every day	12	12%
3–5 times per week	22	22%
1–2 times per week	36	36%
Not at all	30	30%
During the past week, other than your regular job, did you participate in any physical activities or exercises?		
Less than 30 minutes	22	22%
30 minutes to 1 hour	31	31%
More than 1 hour	26	26%
No exercise at all	21	21%
Health insurance		
Yes	47	47%
No Not Sure	39 14	39% 14%
Family doctor		
Yes	12	12%
No	12	12%
Not sure	76	76%
Doctor speaks your language		
Yes	37	37%
No	67	67%
Was there a time in the past 12 months when you needed to see a doctor but could not because of?		
Cost	30	30%
Difficulties in communicating with the doctor	11	11%
Difficulties with transportation	11	11%
Do not trust doctor	9	9%
No time to visit doctor	34	34%
No need to see doctor	45	45%
Other	4	4%
Diagnosed disease		
High blood pressure	10	10%
Type 2 diabetes	7	7%
Cardiovascular heart disease	7	7%
High cholesterol	6	6%
Asthma	4	4%
Chronic kidney disease	Nil	Nil%
Other	10	10%
None	68	68%

**Table 4:** Socio-demographic characteristics of study participants.

Variables	Frequency	Percentage
When you last took antibiotics		
In the last month	14	14%
I the last 6 months	25	25%
In the last year	11	11%
More than year ago	8	8%
Never	8	8%
Cannot remember	34	34%
On that occasion, did you get the antibiotics (or a prescription for them) from a pharmacist, doctor or nurse?		
Yes	49	49%
No	20	20%
Cannot remember	31	31%
On that occasion, did you get the advice/counseling from a doctor, pharmacist or nurse on how to take them?		
Yes	47	47%
No	21	21%
Cannot remember	32	32%
On that occasion, where did you get the antibiotics?		
Medical store or pharmacy	56	56%
Stall or hawker	2	2%
The internet	1	1%
Friend and family	5	5%
I had them saved from a previous time	4	4%
Somewhere/someone else	5	5%
Cannot remember	27	27%

**Table 5:** Reliability test results for knowledge and understanding about antibiotic use and antibiotic resistance.

No.	Questions	Corrected Item-Total Correlation	Cronbach’s Alpha if Item Deleted
**Q1.**	When do you think you should stop taking antibiotics once you’ve begun treatment?	0.056	0.659
**Q3.**	It is okay to use antibiotics that were given to a friend or family member, if they were used to treat the same illness?	0.034	0.660
**Q4.**	It is okay to buy the same antibiotics, or request the same antibiotics from a doctor if I am sick and they helped me get better when I had the same symptoms before?	0.086	0.657
**Q5.**	Do you think these conditions can be treated with antibiotics?	0.013	0.659
**Q6.**	Have you heard of any of the following terms?	0.368	0.668
**Q7.**	Where did you hear about these terms?	0.291	0.651
**Q8.**	Antibiotic resistance occurs when your body becomes resistant to antibiotics and they no longer work as well.	0.015	0.659
**Q8-a**	Many infections are becoming increasingly resistant to treatment by antibiotics.	0.122	0.656
**Q8-b**	If bacteria are resistant to antibiotics, it can be very difficult or impossible to treat the infections they cause.	0.153	0.655
**Q8-c**	Antibiotic resistance is an issue that could affect me or my family.	0.182	0.655
**Q8-d**	Antibiotic resistance is an issue in other countries but not here.	0.077	0.658
**Q8-e**	Antibiotic resistance is only a problem for people who take antibiotics regularly	0.101	0.657
**Q8-f**	Bacteria which are resistant to antibiotics can be spread from person to person.	0.260	0.653
**Q8-g**	Antibiotic-resistant infections could make medical procedures like surgery, organ transplants and cancer treatment much more dangerous.	0.139	0.656
**Q8-h**	Antibiotic resistance occurs when your body becomes resistant to antibiotics and they no longer work as well.	0.015	0.659

**Table 6:** Reliability test results for knowledge and understanding about antibiotic use and antibiotic resistance.

No.	Questions	Corrected Item-Total Correlation	Cronbach’s Alpha if Item Deleted
**Q.6**	**On a scale of 1 to 5 (strongly disagree to strongly agree), how much do you agree with the following statements?**		
Q6-a	People should use antibiotics only when they are prescribed by a doctor or nurse.	0.278	0.647
Q6-b	Farmers should give fewer antibiotics to food-producing animals.	0.062	0.659
Q6-c	People should not keep antibiotics and use them later for other illnesses.	0.134	0.673
Q6-d	Parents should make sure all of their children’s vaccinations are up-to-date.	0.211	0.651
Q6-e	People should wash their hands regularly.	0.173	0.653
Q6-f	Doctors should only prescribe antibiotics when they are needed.	0.148	0.654
Q6-g	Governments should reward the development of new antibiotics.	0.379	0.641
Q6-h	Pharmaceutical companies should develop new antibiotics.	0.179	0.653
**Q.7**	**On a scale of 1 to 5 (strongly disagree to strongly agree), how much do you agree with the following statements?**		
Q7-a	Antibiotic resistance is one of the biggest problems the world faces.	0.202	0.652
Q7-b	Medical experts will solve the problem of antibiotic resistance before it becomes too serious.	0.391	0.644
Q7-c	Everyone needs to take responsibility for using antibiotics responsibly.	0.241	0.650
Q7-d	There are not much people like me can do to stop antibiotic resistance.	0.267	0.649
Q7-e	I am worried about the impact that antibiotic resistance will have on my health, and that of my family.	0.225	0.651
Q7-f	I am not at risk of getting an antibiotic resistant infection, as long as I take my antibiotics correctly.	0.149	0.654
Q7-g	Bacteria which are resistant to antibiotics can be spread from person to person	0.282	0.648
Q7-h	Antibiotic-resistant infections could make medical procedures like surgery, organ transplants and cancer treatment much more dangerous	0.354	0.644
**Q.8**	Are antibiotics widely used in agriculture (including in food-producing animals) in your country?	0.183	0.654

**Table 7:** Reliability test results for knowledge and understanding about antibiotic use and antibiotic resistance.

No.	Questions	Corrected Item-Total Correlation	Cronbach’s Alpha if Item Deleted
**Q.1**	I always use Amoxycillin-clavulanate if ever I have cough or cold, runny nose, either flu or sore throat, diarrhea or fever.	0.596	0.894
**Q.2**	I always use ciprofloxacin if ever I have cough or cold, runny nose, either flu or sore throat, diarrhea or fever.	0.350	0.899
**Q3.**	**On a scale of 1 to 5 (strongly disagree to strongly agree), how much do you agree with the following statements?**		
Q3-a	I generally use antibiotics by myself due to lack of time visiting to the doctor.	0.582	0.894
Q3-b	I generally use antibiotics by myself due to easy availability from pharmacies and chemist shops.	0.612	0.893
Q3-c	I generally use antibiotics by myself due to high cost of visit to the doctors.	0.563	0.894
Q3-d	I generally use antibiotics by myself due to simple signs and symptoms of disease.	0.561	0.894
Q3-e	I generally use antibiotics by myself due to my prior good experience with the same antibiotic.	0.489	0.896
Q3-f	I generally use antibiotics by myself due to lack of trust towards the doctors.	0.507	0.896
Q3-g	I generally use antibiotics by myself if ever I get diarrhea during travelling or holiday abroad.	0.472	0.896
Q3-h	I generally use antibiotics by myself in sore throat/cold/cough immediately to prevent further serious illness.	0.519	0.895

**Table 8:** Association of demographic characteristics of participants with total level of knowledge and understanding scores about antibiotic use and antibiotic resistance.

**Characteristics**	Knowledge and Understanding Scores about Antibiotic Use and Antibiotic Resistance		*p*-Value
	Mean	Median	
**Gender** Male Female	18.7 24.6	19.5 24.5	<0.001 *
**Age** 21–25 years 26–30 years 31–40 years Above 40 years	22.5 23.33 20.7 20.8	22.5 22 21 22.5	0.482
**Ethnicity** Malay Chinese Tamil Others	22.7 22.1 20.9 21.8	23 21 19.5 22	0.004 *
**Education** Primary school High school Certificate/Diploma Undergraduate degree Postgraduate education	10.6 21.4 21 24.5 23.2	10.5 22 22 26 27	<0.001 *
**Household monthly income** Under 3000 Under 3000–5000 Over 5000	20.7 21.5 23.4	22 22 24	0.285
**Marital status** Single Married	22.7 21	23 22	0.241
**Place of birth** Born in Malaysia Not born in Malaysia	22.1 21.5	23 22	0.695
**How long live in Malaysia** Local resident 0–1 Years 2–5 years 6–10 years Above 10 years	22.2 18.3 16.3 23.7 24.6	23 17 17 24.5 24.5	0.003 *
**Occupation** Professional Skilled Labor Manual Labor Administrative Self-employed Home Duties Unemployed Pensioner	25.9 10.3 19.1 24.4 21.3 24.4 23 7	29 17 22 25 21.5 23 22 7	0.016

* Significant, *p*-value < 0.05.

**Table 9:** Association of demographic characteristics with total practice scores towards SMA.

Characteristics	Total Practice Scores for SMA		*p*-Value
	Mean	Median	
**Gender** Male Female	5.7 6.2	6 7	0.004 *
**Age** 21–25 years 26–30 years 31–40 years Above 40 years	6.2 5.7 6.3 5.7	7 6.5 7 6.5	0.761
**Ethnicity** Malay Chinese Tamil Others	5.6 5.9 6.7 6.1	7 6.5 8 7	0.377
**Education** Primary school High school Certificate/Diploma Undergraduate degree Postgraduate education	5.1 6 5.4 6.3 7	4.5 7 5 7 8	0.002 *
**Household monthly income** Under 3000 Under 3000–5000 Over 5000	7 6.6 6.5	8 7 6	0.005 *
**Marital status** Single Married	5.5 6.4	7 7	0.076
**Place of birth** Born in Malaysia Not born in Malaysia	5.6 6.2	7 7	0.181
**How long live in Malaysia** Local resident 0–1 Years 2–5 years 6–10 years Above 10 years	5.7 6.3 6.4 5.9 6.5	7 7 7 6.5 7.5	0.749
**Occupation** Professional Skilled Labor Manual Labor Administrative Self-employed Home Duties Unemployed Pensioner	5.4 7 6 6.2 5 5 6.9 8	6 7 7 8 5 6 8 8	0.097

* Significant, *p*-value < 0.05.
